# Empowering patients for biomarker-informed care: digital education to bridge HER2-low knowledge gaps in metastatic breast cancer

**DOI:** 10.3389/fdgth.2025.1702972

**Published:** 2025-12-11

**Authors:** Heidi C. Ko, Stuti Patel, Rachel E. Ellsworth, Michelle F. Green, Kyle C. Strickland, Jenessa Rossi, Ashima Dua, Maya Said, Amee Sato Dossey, Carole Cuny, Theresa Dunn, Kimberly Weaner, Maria Celeste Ramirez, Cristina Nelson, Linda Bohannon, Jonathan Klein, Marcia Eisenberg, Brian Caveney, Eric A. Severson, Shakti Ramkissoon, Rebecca A. Previs

**Affiliations:** 1 Labcorp, Durham, NC, United States; 2 Department of Pathology, Duke University Medical Center, Duke Cancer Institute, Durham, NC, United States; 3 Outcomes4Me, Boston, MA, United States; 4 Vital Strategic Insights, Indianapolis, IN, United States; 5 Wake Forest Comprehensive Cancer Center and Department of Pathology, Wake Forest School of Medicine, Winston-Salem, NC, United States; 6 Department of Obstetrics & Gynecology, Division of Gynecologic Oncology, Duke University Medical Center, Duke Cancer Institute, Durham, NC, United States

**Keywords:** HER2 low-expression, metastatic breast cancer, patient education, digital health, patient empowerment, health application

## Abstract

**Background:**

The emergence of trastuzumab deruxtecan has led to significant improvement in clinical outcomes for patients with HER2-low metastatic breast cancer, which accounts for approximately half (45%–55%) of breast cancer diagnoses. However, little is known about patients’ awareness of diagnostic testing requirements and treatment implications associated with HER2-low status. This study aims to better understand patients’ knowledge of HER2-low.

**Methods:**

This cross-sectional survey was completed virtually on the Outcomes4Me mobile app, a direct-to-patient digital application that empowers patients to take a proactive approach to their care. Eligible patients included those with Stage IV breast cancer living in the United States. Participants were surveyed on their awareness of their tumor's HER2 biomarker status and willingness to discuss more with their oncologists if their status was unknown. Educational content about HER2 biomarker testing was accessible on the app. Responses were analyzed descriptively and reported in aggregate.

**Results:**

Out of the 527 respondents, 362 met eligibility criteria. Among them, 42% were diagnosed over 5 years ago, 35% had Stage IV disease at diagnosis, 33% received care in a community setting, and 43% had progressed on prior metastatic therapy. The majority (78%, *n* = 284) knew their HER2 status, while 18% (*n* = 64) did not recall it and 4% (*n* = 14) did not respond. Among those aware of their status, 51% were at least somewhat familiar with HER2-low, compared with 23% who were unaware of their HER2 status. Among the patients with known HER2-negative disease (*n* = 152), 74% reported testing within the past year, yet 51% did not recall HER2-low being discussed. Following brief in-app education, 61% of patients with unknown HER2 status at diagnosis (*n* = 64) expressed intent to discuss HER2-low testing with their oncologist.

**Conclusions:**

Knowledge gaps in HER2 biomarker testing persist in patients with metastatic breast cancer. Even for patients with a known HER2 status, many remain unaware of the HER2-low classification. Digital education resources such as the Outcomes4Me app can facilitate patient empowerment and provide targeted education outside of traditional clinical settings, enabling shared decision-making. After receiving a brief education within the app, the majority of patients with an unknown HER2 status expressed willingness to discuss more about HER2 testing with their oncologist.

## Introduction

Breast cancer is a biologically heterogeneous disease characterized by distinct molecular alterations that drive uncontrolled cell growth and invasion (1). Understanding the tumor's unique molecular subtypes based on gene expression patterns, such as luminal A, luminal B, HER2-enriched, and basal-like, is essential for accurate prognostication and therapeutic decisions. For clinical applications, these molecular subtypes can be simplified into three main groups based on key receptor biomarker expression: luminal-like [hormone receptor (HR)-positive], HER2-positive or overexpressed, and triple-negative (lacking HR and HER2 expression) (1, 2).

The luminal-like subtype, characterized by the expression of estrogen and/or progesterone receptors, represents approximately 75% of all breast cancer diagnoses (1). Approximately 15% of breast cancer cases exhibit overexpression of the transmembrane glycoprotein HER2, classifying them as HER2-positive (1, 2). HER2 positivity is defined by an immunohistochemistry (IHC) score of 3+, or a score of 2+ with confirmed *ERBB2* gene amplification via *in situ* hybridization (ISH), indicated by an HER2/CEP17 ratio ≥2.0 and an average *ERBB2* gene copy number ≥4.0 (3). Historically, HER2 is measured on a binary scale—positive (IHC 3+ or 2+ with ISH amplification) or negative (IHC 2+ without ISH amplification or 1+ or 0)—to identify patients likely to benefit from anti-HER2 therapies (3, 4).

However, this treatment paradigm shifted in 2022. Tumors with low HER2 expression, defined as IHC 1+ or 2+ without *ERBB2* gene amplification, have demonstrated clinical benefit from the HER2-targeting antibody–drug conjugate (ADC) trastuzumab deruxtecan (T-DXd) (5). In the Phase III DESTINY-Breast04 trial, T-DXd significantly improved outcomes in patients with HER2-low metastatic breast cancer compared with standard chemotherapy. The trial reported a nearly 50% reduction in the risk of disease progression and a 36% reduction in the risk of death, irrespective of HR status (5). These findings led to the US Food and Drug Administration (FDA) approval of T-DXd for the treatment of advanced HER2-low breast cancer, marking a pivotal shift in the therapeutic landscape.

Given the emerging clinical relevance of HER2-low status and the expansion of HER2 testing modalities in metastatic breast cancer, patients must be adequately informed about the diagnostic and therapeutic implications of HER2 biomarker testing. As oncology care becomes increasingly complex due to evolving treatment paradigms and diverse patient populations, the provision of clear and comprehensible information is essential to facilitate informed decision-making, an integral component of patient-centered care (PCC) throughout the cancer continuum (6, 7). A growing body of research highlights the positive impact of PCC on treatment adherence, chronic disease management, quality of care, and overall health outcomes (7–9). Digital technologies, including mobile applications (apps) and web-based platforms, can effectively support patient education, present guideline-based treatment options, and enhance self-efficacy, ultimately empowering patients to advocate for personalized treatment and supportive care (10–12). Among emerging digital technologies, the rapid rise of mobile health (mHealth) solutions, particularly smartphone apps, has changed the outlook of PCC by providing patients with convenient access to health information, enhancing self-awareness, and facilitating communication with healthcare providers (13–15). Since the introduction of smartphones in 2007, the mHealth app development has surged, with over 300,000 apps available by 2021 (16). Cancer-specific apps have followed this trend as valuable tools for symptom tracking, patient education, and peer support. These apps are widely accepted by patients and demonstrate potential in enhancing patient engagement and facilitating self-management of care (17, 18).

Among the top-rated mHealth solutions, the Outcomes4Me mobile application has been recognized by patients for its high quality and practicality (19). Developed to support individuals with breast cancer, this app aims to improve patient understanding of their diagnosis, facilitate informed navigation of treatment options, and enable ongoing symptom monitoring. A pilot study demonstrated its feasibility and usability, indicating its potential for integration into routine clinical practice to support patient-centered breast cancer care (20). In this study, we aimed to describe patient awareness of HER2 biomarker classifications and analyze engagement with digital educational resources provided through this mobile health application.

## Patients and methods

### Study design and patient population

This cross-sectional survey assessed patient awareness of the HER2 biomarker and the use of Outcomes4Me app resources in empowering patients with metastatic breast cancer to initiate discussions with their providers about HER2 testing. The study was conducted in collaboration between Labcorp (Durham, NC, USA) and Outcomes4Me (Boston, MA, USA). The participants were recruited via targeted push notifications and emails distributed through the Outcomes4Me app between 29 May and 30 November 2023.

Eligible participants included English-speaking women residing in the USA with a confirmed diagnosis of metastatic breast cancer. The study was approved by the Western Copernicus Group Institutional Review Board (WCG IRB, protocol #1340120).

### Implementation of a digital education campaign

**Figure 1:**
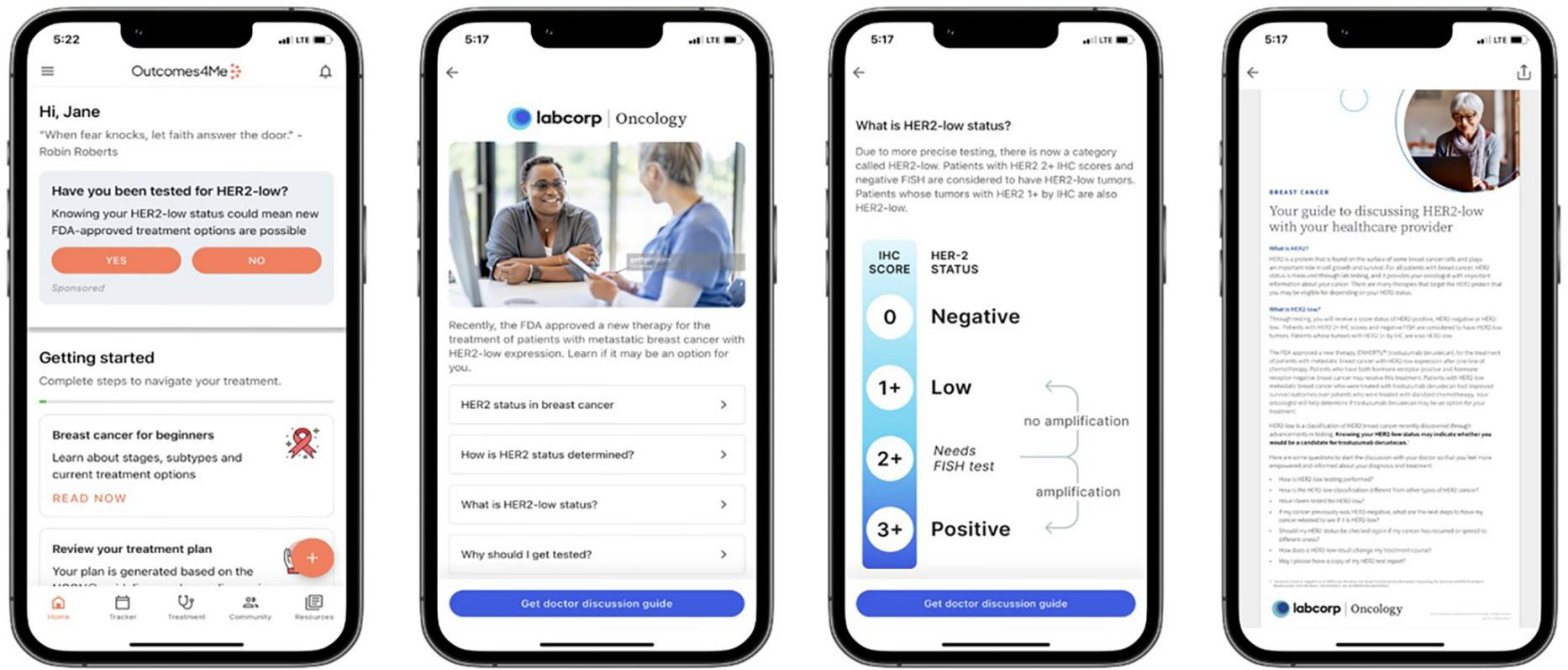
Representative images from the HER2-low educational campaign: Patients were activated through an embedded patient navigation experience supported by push and email. The HER2-low campaign raised awareness of HER2-low testing and provided educational materials. Specifically, for patients with metastatic breast cancer, the campaign enabled them to download a doctor discussion guide. Reprinted with permission from Outcomes4Me Inc, “Digital Education Campaign on HER2 Testing and Discussion Guide with Labcorp” Outcomes4Me Digital App, https://outcomes4me.com.

A structured digital campaign was developed to enhance awareness and understanding of the HER2 biomarker among patients with metastatic breast cancer. Educational content was codeveloped with input from oncologists, patient advocates, and software designers to ensure clarity, accessibility, and clinical relevance. The campaign featured a multicomponent educational module within the Outcomes4Me app, including an interactive doctor discussion guide, a patient-friendly webinar titled “What is HER2-low and Why Should I Get Tested?”, and layered modules explaining HER2 biology, classification, testing, and treatment implications (Figure 1). Behavioral nudges, such as reminders, prompts within the app feed, push notifications, and follow-up emails, were incorporated to encourage patient engagement and facilitate provider discussions.

### Survey design and data collection

The participants were invited to complete a baseline survey (see Supplementary material) upon first exposure to the educational campaign. The survey was developed collaboratively with oncologists, patient advocates, and designers to ensure clarity and clinical relevance. It comprises three main sections:

1. **Demographic and clinical information**: This section captured patient characteristics, including age, geographic location (city and zip code), race/ethnicity, stage at diagnosis, treatment history, and practice setting of the treating oncologist.
2. **Awareness and recall of biomarker testing**: In this section, a series of questions evaluated awareness of HER2 testing and HER2-low classification, recall of oncologist discussions, timing of HER2 testing, and communication of results. Responses were primarily categorical (e.g., “very aware,” “somewhat aware,” and “not at all aware”) and included multiple-choice formats.
3. **Educational needs and intent to act**: This section assessed interest in clinical trials, preferred educational resources, and the likelihood of discussing HER2 testing with an oncologist after reviewing definitions. Likert-type scales were used for intent-related questions (e.g., “very likely” to “not at all likely”).

All surveys were administered electronically through the Outcomes4Me mobile application. Participation was voluntary, with no incentives provided, and all respondents who completed the baseline survey were included in the analysis. Although the instrument underwent internal review for content validity, formal psychometric validation (e.g., reliability testing and comprehensibility assessment) was not performed prior to survey deployment, which is acknowledged as a study limitation.

### Statistical analysis

Descriptive statistics were used to evaluate participant demographics, clinical history, awareness levels, and in-app behaviors. Data were reported in aggregate and stratified by HER2 status where appropriate. Engagement metrics, including reach, content views, and deep engagement, were collected via backend analytics from the Outcomes4Me platform. The primary objective of the study was to characterize patient awareness of HER2 testing and assess engagement outcomes, including their willingness to participate in discussions with healthcare providers following education through the campaign.

## Results

### Campaign performance and user engagement

Between 29 May and 30 November 2023, a HER2-low educational campaign launched via the Outcomes4Me mobile app and reached 10,638 unique patients with breast cancer. Of these patients, 7,749 clicked through to view the campaign content, and 2,034 demonstrated deep engagement by taking actions such as exploring supplementary materials, downloading the doctor discussion guide, or activating calls to action. Monthly engagement remained steady throughout the 6-month campaign, with peak activity observed in June and July 2023.

### Patient characteristics

Among the 527 total respondents who completed the baseline survey, 362 women met the inclusion criteria, such as living in the USA and having metastatic breast cancer. Demographic and clinical data for these individuals are shown in Table 1. The most common age group was between 60 and 69 years (30%), followed by 50–59 (25%) and 40–49 (15%). Based on racial and ethnic self-identification, 64% of individuals identified as White, 8% as Black or African American, and 4% as Hispanic or Latino. In terms of clinical and treatment characteristics, 152 (42%) patients were diagnosed at least 5 years prior to taking the survey, 128 (35%) were diagnosed with Stage IV breast cancer, 119 (33%) received treatment within a community setting, and 157 (43%) were receiving treatment for metastatic cancer that had progressed on at least one line of therapy.

**Table 1:** Clinico-demographics of survey respondents during a HER2-low educational campaign launched via the Outcomes4Me mobile application.

Survey question	Number of eligible participants (*n* = 362, %)
When were you first diagnosed with breast cancer?	
No response	2, 0.01%
<6 months ago	32, 9%
6 months–1 year ago	36, 10%
1–2 years ago	61, 17%
2–5 years ago	79, 22%
5+ years ago	152, 42%
What practice setting is your oncologist located in?	
Community practice	119, 33%
Academic cancer center	101, 28%
Veteran's Affairs	64, 18%
Other	54, 15%
No response	20, 5%
Do not know	4, 1%
Stage at initial diagnosis	
Stage I	83, 23%
Stage II	68, 19%
Stage III	61, 17%
Stage IV/metastatic	128, 35%
No response	3, 1%
Do not know/do not recall	19, 5%
What is your race and/or ethnicity?	
Multiple races	17, 5%
Hispanic, Latino, or Spanish	14, 4%
Black or African American	28, 8%
White	232, 64%
Prefer not to answer	8, 2%
No response	63, 17%
Which of the following best describes where you are in your treatment (Rx) journey?	
My cancer has returned, but I have chosen not to receive Rx	8, 2%
After diagnosis, but before deciding on Rx	19, 5%
Completed Rx and received post-Rx follow-up	33, 9%
On Rx	93, 26%
Received Rx for metastatic cancer that has progressed at least once	157, 43%
Other	21, 6%
Do not know/do not recall	8, 2%
No response	23, 7%

### Awareness and recall of HER2 status at diagnosis

**Figure 2:**
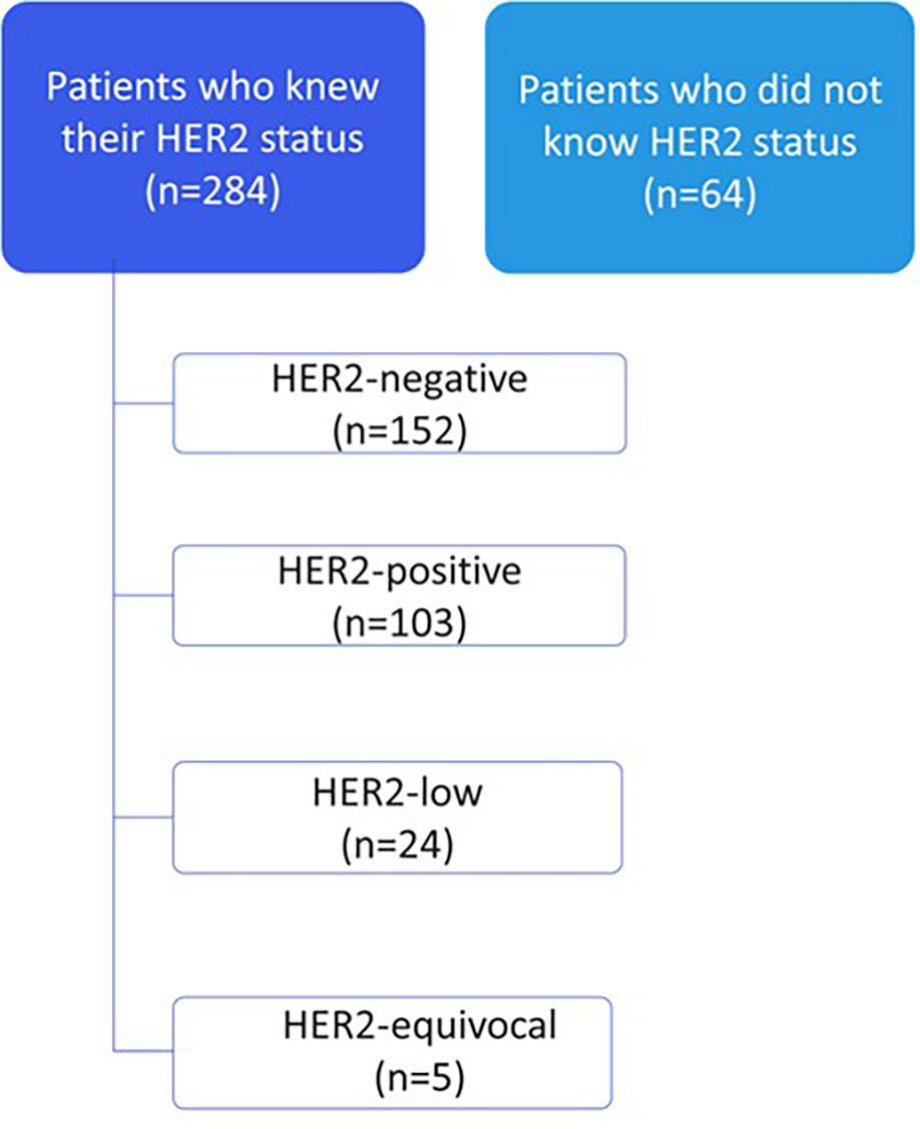
Patient awareness of HER2 status at diagnosis (*n* = 348).

Among the 362 eligible patients with metastatic breast cancer, 348 completed the survey, of whom 284 reported knowing their HER2 status at diagnosis, while 64 reported they did not know or recall their HER2 status. A total of 14 patients did not respond to follow-up questions and were excluded from subsequent analyses. Among patients with known HER2 status, 152 had HER2-negative disease; 103 and 24 patients had HER2-positive and HER2-low, respectively; and 5 patients had equivocal HER2 results (Figure 2).

**Figure 3:**
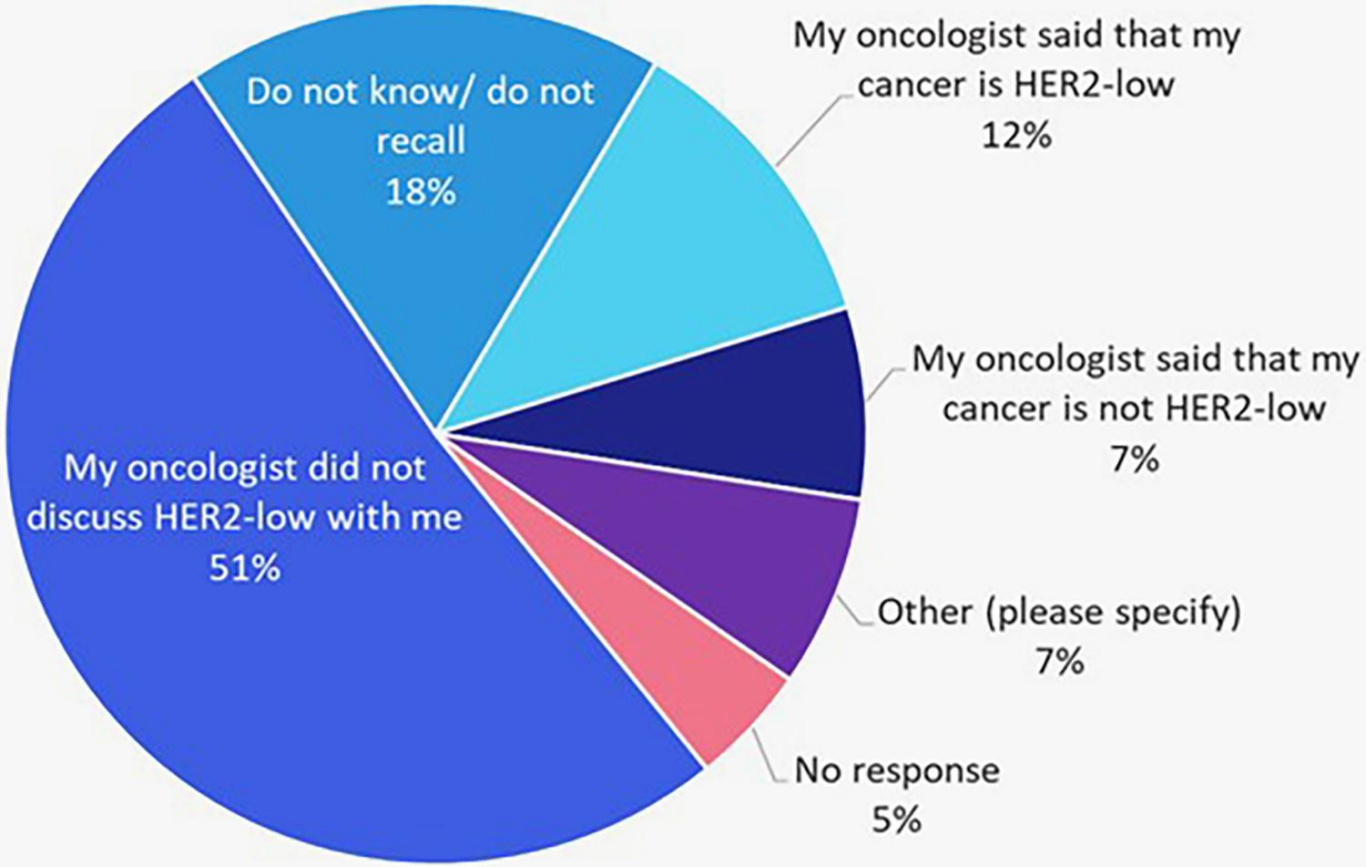
Recall of whether HER2-low as a possible result was discussed by treating oncologists among patients with recently (within the past year) diagnosed HER2-negative cancers (*n* = 112).

Of the 152 patients with HER2-negative disease, 112 were diagnosed within the past year. Among them, 51% reported no discussion of HER2-low status with their oncologist. Only 12% were told their cancer was HER2-low, 7% were told it was not, 18% were unsure or couldn’t recall, and 5% did not respond (Figure 3).

### Awareness of HER2-low classification

**Figure 4:**
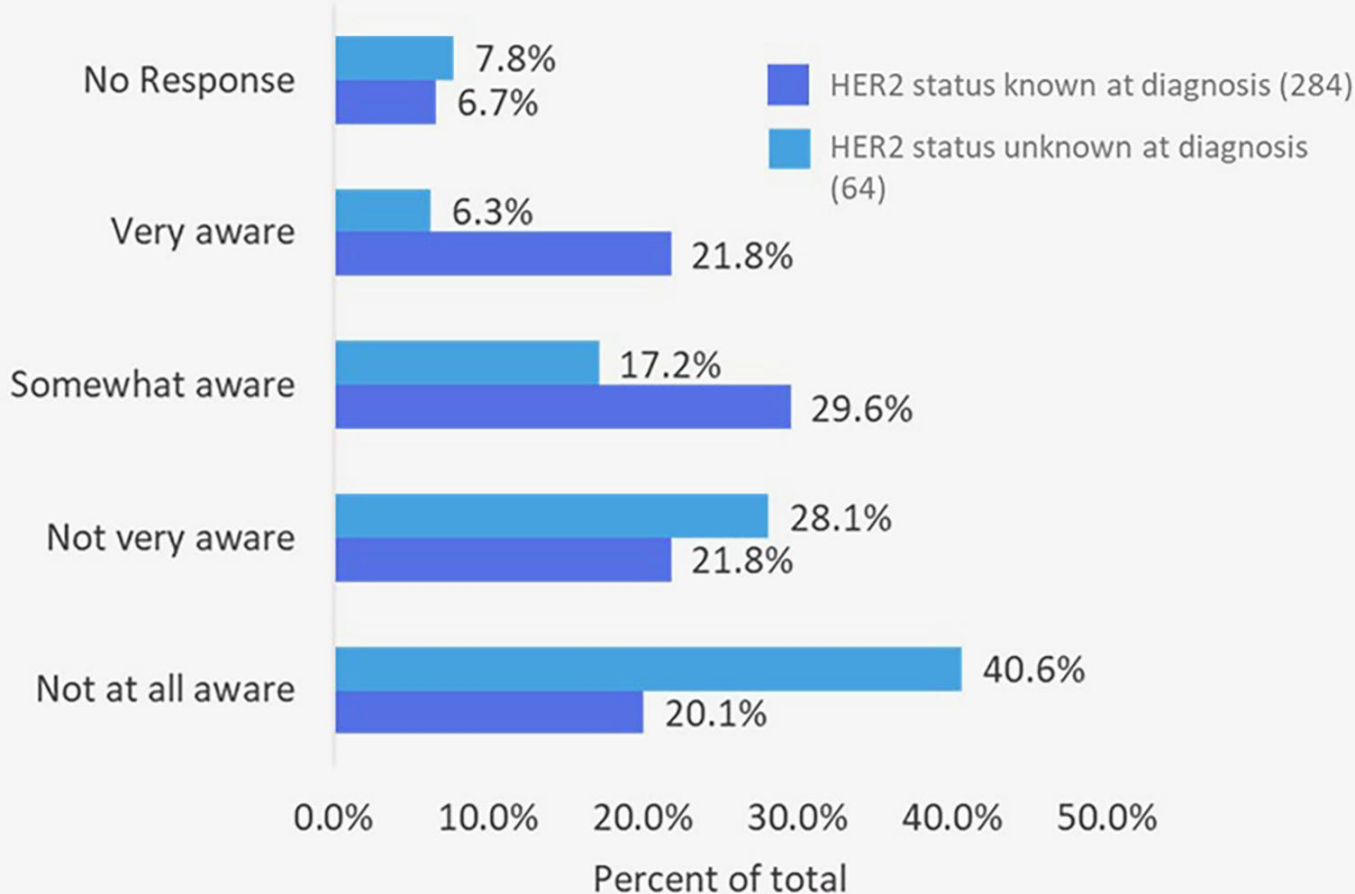
Patient self-reported awareness of HER2-low classification in breast cancer (*n* = 348 who completed the survey).

Awareness of HER2 testing varied by patients' knowledge of their HER2 status at diagnosis. Among the 284 patients who knew their status, 51% were at least somewhat aware of HER2-low, compared with only 23% of the 64 patients who did not know their status where (Figure 4). This highlights a notable awareness gap in understanding of a recently recognized biomarker now associated with an FDA-approved treatment option.

### Effect of brief digital education on patient empowerment and intent to discuss HER2-low testing

**Figure 5:**
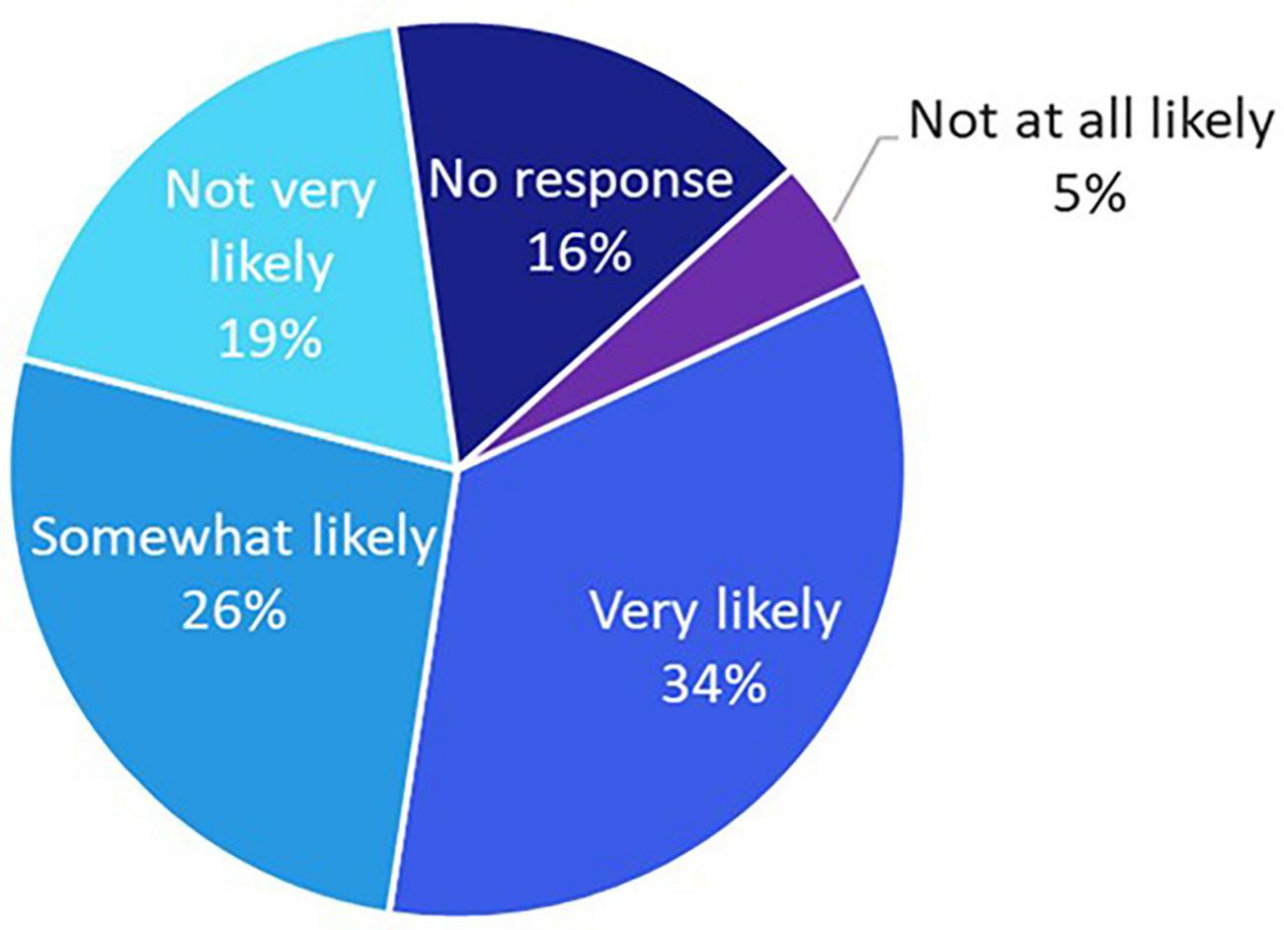
The likelihood of patients with an unknown biomarker status (*n* = 64) to talk to their oncologist about HER2-low after brief education about HER2 testing was provided within the app.

To evaluate the impact of targeted digital education on patient empowerment, a subset of participants with unknown HER2 status (*n* = 64) received brief in-app educational content. Following the intervention, 60% reported being “very likely” or “somewhat likely” to initiate a conversation with their oncologist about HER2-low testing (Figure 5).

## Discussion

This study highlights the ongoing gaps in patient awareness of HER2 biomarker classifications, particularly the emerging HER2-low subtype, among individuals with metastatic breast cancer. Although most respondents knew their HER2 status at diagnosis, 18% were unaware of HER2 testing. Among those with HER2-negative disease diagnosed within the past year, 51% of HER2-negative patients did not recall any discussion of HER2-low status, suggesting that biomarker testing may occur without adequate patient education or shared decision-making. These findings align with prior research where 30% of patients at a comprehensive cancer center did not recall undergoing genomic testing (21), and in a broader survey, 50% of patients reported low familiarity with biomarker testing despite high testing rates, with 75% expressing unmet informational needs (22). Our results reinforce the need for patient-facing educational strategies beyond traditional clinical encounters, particularly as HER2-low is now a clinically actionable subtype with approved therapies such as T-DXd (5, 23).

Digital platforms have emerged as promising tools to bridge this gap in knowledge. Kirsch et al. (10) performed a comprehensive literature review across 25 studies evaluating the utilization and effects of digital health platforms for breast cancer care. The findings demonstrated that mobile apps and web-based tools not only provided patient education and symptom monitoring but also improved patient outcomes, including improved quality of life and reduced psychological distress (10). Similarly, a recent systematic literature review of 39 studies highlights how digital health applications can significantly improve patient education and empowerment among individuals with cancer (24). By delivering tailored educational content, real-time symptom tracking, and support tools, these apps enhance patients' understanding of their health status and available treatment options. The findings underscore that such platforms not only increase health literacy but also foster active participation in care decisions, leading to improved patient confidence and autonomy (24). Moreover, our study demonstrated that a brief educational prompt on HER2 testing delivered through the Outcomes4Me app, along with user interaction, significantly improved patient engagement. Notably, over half of the users who were initially unaware of their HER2 status reported a strong intention to discuss HER2-low with their oncologist after reviewing the educational content through the Outcomes4me digital platform.

Our study has several limitations. First, although survey questions and educational content were developed collaboratively with oncologists, patient advocates, and designers to ensure clarity and relevance, formal psychometric validation (e.g., reliability testing and comprehensibility assessment) was not conducted prior to deployment. This may affect the reliability and generalizability of findings. Second, the participant population lacked demographic diversity, with 64% identifying as White and limited representation of Black, Hispanic, and other minority groups, which restricts applicability to broader patient populations. Third, all data were self-reported, introducing potential recall and social desirability bias that may influence the accuracy of HER2 status awareness and recall of oncologist discussions. Fourth, engagement with educational modules was low; only 34 participants completed the follow-up survey, and just 24 interacted with all available content, reducing statistical power and robustness of impact analyses. Finally, while intent to discuss HER2 testing was measured, we did not assess whether this translated into actual biomarker testing or treatment changes, nor did we link engagement to clinical outcomes such as therapy alignment.

Future research should incorporate structured validation processes, evaluate strategies to integrate digital education equitably into clinical care, and measure whether engagement influences clinical outcomes such as biomarker testing rates and treatment alignment. Approaches such as multilingual content, culturally tailored messaging, personalized reminders, and provider-led follow-up may enhance reach and effectiveness. Collaborations with cancer centers, community organizations, and advocacy groups will be critical to ensure inclusive access and support shared decision-making across diverse populations.

## Conclusion

This study identifies a gap in patient understanding of HER2-low as a clinically relevant biomarker in metastatic breast cancer. Despite general awareness of HER2 status, many patients lacked knowledge of HER2-low implications, potentially delaying access to effective therapies. The Outcomes4Me digital platform demonstrates a scalable approach to addressing this gap; brief, targeted education via the app increased patient intent to discuss HER2 status with oncologists, particularly among those previously unaware. As HER2-low testing gains importance in precision oncology, equipping patients with the knowledge and tools to advocate for biomarker-informed care is critical. While further efforts are needed to reach underserved populations and assess downstream clinical impact, this study provides proof-of-concept for digital interventions in advancing personalized and equitable cancer care.

## Data Availability

The datasets generated and/or analyzed during the current study are proprietary. Raw data supporting the findings of this study may be made available upon reasonable request.
