# Supplementary material for: Empowering patients for biomarker-informed care: digital education to bridge HER2-low knowledge gaps in metastatic breast cancer
Source: Front Digit Health. 2025 Dec 11;7:1702972. doi: 10.3389/fdgth.2025.1702972 (PMC12738299; doi:10.3389/fdgth.2025.1702972)
Supplement: Supplementary file 1 [file Datasheet1.pdf]

Understanding of HER2 low testing in metastatic breast cancer

We are conducting this study to evaluate the awareness and understanding of HER2-low testing in metastatic breast cancer patients. We'll publish the results at an upcoming conference. The survey takes up to 10 minutes to complete.

Understanding of HER2 low testing in metastatic breast cancer

* 1. Do you currently live in the United States?

- ◯ Yes
- ◯ No

Understanding of HER2 low testing in metastatic breast cancer

* 2. Are you currently or have you ever been diagnosed with metastatic (stage IV) breast cancer?

- ◯ Yes
- ◯ No

Understanding of HER2 low testing in metastatic breast cancer

* 3. When were you first diagnosed with breast cancer?

- ◯ Less than 6 months ago
- ◯ 6 months – 1 year ago
- ◯ 1 year – 2 years ago
- ◯ 2 years – 5 years ago
- ◯ 5+ years ago

Understanding of HER2 low testing in metastatic breast cancer

* 4. At initial diagnosis, what stage of breast cancer were you diagnosed with?

- Stage I
- Stage II
- Stage III
- Metastatic-Stage IV
- Do not know/do not recall

Understanding of HER2 low testing in metastatic breast cancer

* 5. When your cancer was first diagnosed, it was tested for “hormone receptors” called estrogen receptor (ER) and progesterone receptor (PR). Which category best describes your breast cancer?

- Hormone-receptor positive (ER positive and PR positive)
- Hormone-receptor positive (ER negative and PR positive)
- Hormone-receptor positive (ER positive and PR negative)
- Hormone-receptor negative (ER negative and PR negative)
- Do not know/do not recall

Understanding of HER2 low testing in metastatic breast cancer

* 6. When your cancer was first diagnosed, it was tested for the absence or presence of a protein called HER2. Which category best describes your breast cancer?

- HER2-positive
- HER2-negative
- HER2-low
- HER2-equivocal
- Do not know/do not recall

Understanding of HER2 low testing in metastatic breast cancer

* 7. In what practice setting is your oncologist located?

- ○ Academic comprehensive cancer center (e.g Memorial Sloan, Dana Farber)
- ○ Community practice (e.g. Cancer Treatment Centers of America)
- ○ US Veterans Affairs clinic
- ○ Do not know
- ○ Other (please specify)

Understanding of HER2 low testing in metastatic breast cancer

* 8. Which of the following choices best describes where you are in your cancer treatment journey?

- ○ After diagnosis but before deciding on treatment
- ○ On treatment
- ○ Completed treatment and receiving post-treatment follow-up or in remission
- ○ My cancer has returned, but I have chosen not to receive treatment
- ○ Receiving treatment for metastatic cancer that has progressed at least once
- ○ Do not know/ do not recall
- ○ Other (please specify)

Understanding of HER2 low testing in metastatic breast cancer

* 9. What type of treatment(s) have you received for your breast cancer? Please select all that apply.

- [ ] Chemotherapy
- [ ] Surgery
- [ ] Radiation therapy
- [ ] Endocrine or hormonal therapy
- [ ] Anti-HER2 therapy
- [ ] Immunotherapy
- [ ] Targeted therapy
- [ ] No treatment
- [ ] Other (please specify)

Understanding of HER2 low testing in metastatic breast cancer

* 10. Where are you getting your information regarding breast cancer and treatments? Please select all that apply.

- [ ] Oncologist
- [ ] Family members/friends
- [ ] Internet searching
- [ ] Social media sites
- [ ] Advocacy organizations
- [ ] Outcomes4Me
- [ ] Other (please specify)

Understanding of HER2 low testing in metastatic breast cancer

* 11. How aware are you of HER2 testing in breast cancer?

- ○ Very aware
- ○ Somewhat aware
- ○ Not very aware
- ○ Not at all aware

Understanding of HER2 low testing in metastatic breast cancer

* 12. Have you discussed your HER2 test results with your oncologist?

- ◯ Yes
- ◯ No
- ◯ Do not know

Understanding of HER2 low testing in metastatic breast cancer

* 13. How aware are you of HER2-low classification in breast cancer?

- ◯ Very aware
- ◯ Somewhat aware
- ◯ Not very aware
- ◯ Not at all aware

Understanding of HER2 low testing in metastatic breast cancer

* 14. Have you discussed HER2-low testing with your oncologist?

- ◯ Yes
- ◯ No
- ◯ Do not know

Understanding of HER2 low testing in metastatic breast cancer

* 15. When was the most recent time you had HER2 testing?

- ◯ Within the last year
- ◯ Between 1 and 5 years ago
- ◯ Between 5 and 10 years ago
- ◯ More than 10 years ago

Understanding of HER2 low testing in metastatic breast cancer

* 16. How did you get the results of your HER2 testing? Please select all that apply.

- [ ] My healthcare provider gave them to me.
- [ ] I viewed the report on patient portal
- [ ] I received testing but do not recall whether I received results
- [ ] Do not know
- [ ] Other (please specify)

Understanding of HER2 low testing in metastatic breast cancer

* 17. When was the most recent time you had HER2 testing?

- ○ Within the last year
- ○ Between 1 and 5 years ago
- ○ Between 5 and 10 years ago
- ○ More than 10 years ago

Understanding of HER2 low testing in metastatic breast cancer

* 18. Has your oncologist shared the results of your HER2 testing with you?

- ○ Yes
- ○ No
- ○ Don’t know if I had testing

Understanding of HER2 low testing in metastatic breast cancer

* 19. What were the results of your HER2 testing? Please select all that apply.

- [ ] I found out that I had HER2-positive breast cancer
- [ ] I found out that I had HER2-negative breast cancer
- [ ] I found out that I had HER2-low breast cancer
- [ ] Do not know/ do not recall
- [ ] Other (please specify)

Understanding of HER2 low testing in metastatic breast cancer

* 20. If your breast cancer is HER2-negative, has your oncologist discussed HER2-low as a possibility?

- ○ My oncologist said that my cancer is HER2-low
- ○ My oncologist said that my cancer is not HER2-low
- ○ My oncologist did not discuss HER2-low with me
- ○ Do not know/ do not recall
- ○ Other (please specify)

Understanding of HER2 low testing in metastatic breast cancer

* 21. Are you interested in information about participating in a clinical trial?

- ○ Yes
- ○ No
- ○ Do not know

Understanding of HER2 low testing in metastatic breast cancer

* 22. How would you like to learn more about clinical trials?

- ☐ I want to discuss the clinical trial opportunities relevant to my cancer with my oncologist
- ☐ I want to do internet searching on sites like clinicaltrials.gov.
- ☐ I am already enrolled in a clinical trial
- ☐ Other (please specify)

Understanding of HER2 low testing in metastatic breast cancer

* 23. Are you interested in information about HER2 testing?

- ○ Yes
- ○ No

Understanding of HER2 low testing in metastatic breast cancer

* 24. What informational materials would be the most helpful to you?

- [ ] Educational content
- [ ] Doctor discussion guides
- [ ] Webinar
- [ ] Discussion with the nurse
- [ ] Other (please specify)

Understanding of HER2 low testing in metastatic breast cancer

* 25. Which city do you live in?

Understanding of HER2 low testing in metastatic breast cancer

* 26. What is your zip code?

Understanding of HER2 low testing in metastatic breast cancer

27. What is your race and/or ethnicity? (click all that applies) (optional)

- ☐ White
- ☐ Black or African-American
- ☐ Asian or Asian Indian
- ☐ American Indian or Alaskan Native
- ☐ Hispanic, Latino, or Spanish
- ☐ Middle Eastern or North African
- ☐ Native Hawaiian or Other Pacific Islander
- ☐ Prefer not to answer
- ☐ Other (please specify)

Understanding of HER2 low testing in metastatic breast cancer

28. Any other comments you’d like to share? (optional)

* 29. Please enter your email so we can send you details on how to sign up for Outcomes4Me. We are the only direct-to-patient platform that integrates with the NCCN Clinical Practice Guidelines in Oncology (NCCN Guidelines®), from the not-for-profit alliance of 32 leading cancer centers.

We gather treatment recommendations typically meant for oncologists and use Artificial Intelligence (AI) to translate that information so you can understand it, putting you in control. With this knowledge, you can feel empowered to make the best medical decisions with your care team.

* 30. In what practice setting is your oncologist located?

- Academic comprehensive cancer center (e.g. Memorial Sloan, Dana Farber)
- Community practice (e.g. Cancer Treatment Centers of America)
- US Veterans Affairs clinic
- Do not know
- Other (please specify)

Understanding of HER2 low testing in metastatic breast cancer

* 31. Which of the following choices best describes where you are in your cancer treatment journey?

- After diagnosis but before deciding on treatment
- On treatment
- Completed treatment and receiving post-treatment follow-up or in remission
- My cancer has returned, but I have chosen not to receive treatment
- Receiving treatment for metastatic cancer that has progressed at least once
- Do not know/do not recall
- Other (please specify)

Understanding of HER2 low testing in metastatic breast cancer

* 32. What type of treatment(s) have you received for your breast cancer? Please select all that apply.

- [ ] Chemotherapy
- [ ] Surgery
- [ ] Radiation therapy
- [ ] Endocrine or hormonal therapy
- [ ] Anti-HER2 therapy
- [ ] Immunotherapy
- [ ] Targeted therapy
- [ ] No treatment
- [ ] Other (please specify)

Understanding of HER2 low testing in metastatic breast cancer

* 33. Where are you getting your information regarding breast cancer and treatments? Please select all that apply.

- [ ] Oncologist
- [ ] Family members/friends
- [ ] Internet searching
- [ ] Social media sites
- [ ] Advocacy organizations
- [ ] Outcomes4Me
- [ ] Other (please specify)

Understanding of HER2 low testing in metastatic breast cancer

* 34. How aware are you of HER2 testing in breast cancer?

- ( ) Very aware
- ( ) Somewhat aware
- ( ) Not very aware
- ( ) Not at all aware

Understanding of HER2 low testing in metastatic breast cancer

* 35. Have you discussed your HER2 test results with your oncologist?

- ◯ Yes
- ◯ No
- ◯ Do not know

Understanding of HER2 low testing in metastatic breast cancer

* 36. How aware are you of HER2-low classification in breast cancer?

- ◯ Very aware
- ◯ Somewhat aware
- ◯ Not very aware
- ◯ Not at all aware

Understanding of HER2 low testing in metastatic breast cancer

* 37. Have you discussed HER2-low testing with your oncologist?

- ◯ Yes
- ◯ No
- ◯ Do not know

Understanding of HER2 low testing in metastatic breast cancer

HER2 testing: In breast cancer, having too much HER2 protein can lead to uncontrolled cancer cell growth and aggressive cancer behavior. However, HER2 can be treated with specific treatment that targets its production. It is, therefore, important to have your healthcare team check your HER2 status after a breast cancer diagnosis so that you and your oncologist can choose a more personalized treatment plan for your cancer if available. This test is typically done using a tissue sample from your tumor, which is sent to the lab for special testing called immunohistochemistry (IHC). IHC measures how much HER2 protein is on the surface of your cancer cell. In the past, any cancer with an IHC score lower than 2 was considered HER2-negative.

HER2-low classification: Now, there is a new classification of HER2 called HER2-low. A breast cancer with an IHC score between 1 and 2 is called HER2-low. Based on this

new classification, there is now a new way to target and treat HER2-low metastatic breast cancer.

HER2-negative classification: Tumors without HER2 proteins will have an IHC score of 0. Your doctor will not recommend HER2-targeted therapy as part of your treatment.

HER2-positive classification: Tumors with HER2 proteins will have an IHC score between 2 and 3. These cancers may be treated with HER2-targeted therapies in addition to other treatments

Understanding of HER2 low testing in metastatic breast cancer

* 38. Based on the definitions you just read, how strongly do you feel about discussing your HER2 results with your oncologist?

- ◯ Very strong
- ◯ Somewhat strong
- ◯ Neutral
- ◯ Do not think it’s important

Understanding of HER2 low testing in metastatic breast cancer

* 39. How likely are you to talk to your oncologist about getting HER2-low testing?

- ◯ Very likely
- ◯ Somewhat likely
- ◯ Not very likely
- ◯ Not at all likely

Understanding of HER2 low testing in metastatic breast cancer

* 40. Which city do you live in?

[ ]

Understanding of HER2 low testing in metastatic breast cancer

* 41. What is your zip Code?

Understanding of HER2 low testing in metastatic breast cancer

42. What is your race and/or ethnicity? (click all that applies) (optional)

- [ ] White
- [ ] Black or African-American
- [ ] Asian or Asian Indian
- [ ] American Indian or Alaskan Native
- [ ] Hispanic, Latino, or Spanish
- [ ] Middle Eastern or North African
- [ ] Native Hawaiian or Other Pacific Islander
- [ ] Prefer not to answer
- [ ] Other (please specify)

Understanding of HER2 low testing in metastatic breast cancer

43. Any other comments you'd like to share? (optional)

Understanding of HER2 low testing in metastatic breast cancer

* 44. Please enter your email so we can send you details on how to sign up for Outcomes4Me. We are the only direct-to-patient platform that integrates with the NCCN Clinical Practice Guidelines in Oncology (NCCN Guidelines®), from the not-for-profit alliance of 32 leading cancer centers.

We gather treatment recommendations typically meant for oncologists and use Artificial Intelligence (AI) to translate that information so you can understand it, putting you in control. With this knowledge, you can feel empowered to make the best medical decisions with your care team.
